# Influence of Confining Element Stiffness on the In-Plane Seismic Performance of Confined Masonry Walls

**DOI:** 10.3390/ma17133100

**Published:** 2024-06-25

**Authors:** Muhammad Mubashir Ajmal, Asad Ullah Qazi, Ali Ahmed, Ubaid Ahmad Mughal, Syed Minhaj Saleem Kazmi, Muhammad Junaid Munir

**Affiliations:** 1 Department of Civil, Environmental and Transportation Systems, University of Sargodha, Sargodha 40100, Pakistan; 2 Department of Civil Engineering, University of Engineering and Technology, Lahore 54000, Pakistan; 3 Guangdong Provincial Key Laboratory of Durability for Marine Civil Engineering, Shenzhen University, Shenzhen 518060, China

**Keywords:** confined masonry, finite element analysis (FEA), reverse cyclic loading, reinforcement ratio, confining element size, ANSYS

## Abstract

Confined masonry (CM) construction is being increasingly adopted for its cost-effectiveness and simplicity, particularly in seismic zones. Despite its known benefits, limited research exists on how the stiffness of confining elements influences the in-plane behavior of CM. This study conducted a comprehensive parametric analysis using experimentally validated numerical models of single-wythe, squat CM wall panels under quasi-static reverse cyclic loading. Various cross-sections and reinforcement ratios were examined to assess the impact of the confining element stiffness on the deformation response, the cracking mechanism, and the hysteretic behavior. The key findings included the observation of symmetrical hysteresis in experimental CM panels under cyclic loading, with a peak lateral strength of 114.3 kN and 108.5 kN in push-and-pull load cycles against 1.7% and 1.3% drift indexes, respectively. A finite element (FE) model was developed based on a simplified micro-modeling approach, demonstrating a maximum discrepancy of 2.6% in the peak lateral load strength and 5.4% in the initial stiffness compared to the experimental results. The parametric study revealed significant improvements in the initial stiffness and seismic strength with increased depth and reinforcement in the confining elements. For instance, a 35% increase in the lateral strength was observed when the depth of the confining columns was augmented from 150 mm to 300 mm. Similarly, increasing the steel reinforcement percentage from 0.17% to 0.78% resulted in a 16.5% enhancement in the seismic strength. These findings highlight the critical role of the stiffness of confining elements in enhancing the seismic performance of CM walls. This study provides valuable design insights for optimizing CM construction in seismic-prone areas, particularly regarding the effects of confining element dimensions and reinforcement ratios on the structural resilience.

## 1. Introduction

Masonry, which has historically been a primary construction method due to its affordability and simplicity [1,2,3], has been used for millennia in significant structures such as the Tower of Babylon and the Great Wall of China [4]. Currently, over half of the global population resides in brick masonry homes [5]. However, in seismic zones, this system presents risks. Unreinforced masonry, while effective under gravity loads, lacks sufficient shear and tensile strength, posing challenges in seismic resilience [6,7]. Despite these drawbacks, the widespread use of masonry persists in earthquake-prone areas, necessitating a reassessment of its seismic vulnerability. Historical seismic events have repeatedly demonstrated the inadequate performance of such structures, often leading to considerable damage and the loss of life, as has been experienced in China, Japan, Iran, Indonesia, India, Italy, and Pakistan [8,9].

The AJK earthquake in October 2005, a catastrophic event in Pakistan, marked a turning point in the approach to masonry structures in seismic regions. This disaster resulted in over 73,000 fatalities, injured 70,000, left 2.8 million people homeless, and destroyed more than 450,000 buildings [10]. The primary factors contributing to this extensive devastation were identified as sub-standard construction materials, inadequate interlocking between in-plane and out-of-plane walls, and prevalent non-engineered building practices [11]. The sheer scale of this tragedy exposed the urgent need for reform in construction methodologies, particularly in regions susceptible to seismic activity.

In response, there has been a significant shift towards confined masonry construction, a technique that offers a higher lateral stiffness and a better resistance to seismic forces. Originating after the earthquakes in Messina, Italy (1908), and Talca, Chile (1929), confined masonry is characterized by the use of tie-beams and tie-columns that are added after the construction of the masonry wall, enhancing both the in-plane and out-of-plane strength. This method has been studied extensively due to its distinct advantages over traditional construction techniques, particularly in seismic regions.

Jain et al. [12] conducted comprehensive analyses demonstrating the superior seismic performance of confined masonry structures compared to conventional masonry and reinforced concrete frames. Marques et al. [13] highlighted the structural benefits of confined masonry, noting its ability to significantly reduce the risk of collapse during seismic events due to the integration of reinforced concrete tie-beams and tie-columns. This integration enhances the ductility and energy dissipation capacity of masonry walls.

Borah et al. [14] and Brzev [15] provided empirical evidence and theoretical analyses confirming the improved lateral strength and stiffness of confined masonry structures. Their studies emphasized the method’s efficacy in distributing seismic forces more uniformly, thereby reducing the damage concentration in any single structural component. Erberik et al. [16] further elaborated on the mechanical behavior of confined masonry under dynamic loading, revealing that this construction technique offers a balanced combination of strength and flexibility.

Gouveia et al. [17] conducted detailed finite element modeling to compare confined masonry with traditional infill masonry frames, concluding that the former exhibits superior seismic resilience. Schacher [18] provided case studies from various earthquake-prone regions, showcasing the practical implementation and performance of confined masonry in real-world scenarios. Singhal et al. [19] analyzed post-earthquake damage data, underscoring the reduced vulnerability of confined masonry buildings.

The People’s Republic of China National Standard [20] and Tomaževič et al. [21] offered regulatory perspectives and guidelines, reinforcing the adoption of confined masonry in building codes due to its proven effectiveness. Tu et al. [22] explored the construction process and the role of craftsmanship in ensuring the structural integrity of confined masonry. Finally, Koji Yoshimura et al. [23] investigated the environmental and economic benefits of confined masonry, noting its cost-effectiveness and sustainability.

The adoption of confined masonry is not limited to Pakistan, but is recognized globally as an economical and resilient solution for earthquake-prone areas, suitable for buildings of up to six stories. Several guidelines have emerged in recent years to set the fundamental details of confined masonry. A few countries have established codes for confined masonry design, for instance, China [20], Europe [24], Costa Rica [25], Colombia [26], Argentina [27], Chile [28], Peru [29], and Mexico [30]. Also, the building code of Pakistan is in a development phase for confined masonry [31]. However, despite the development and implementation of numerous international standards and codes for confined masonry design, significant disparities in these guidelines persist. These include variations in the provisions for in-plane and out-of-plane seismic strength and the design and detailing of confining elements [32,33,34,35,36,37].

Confined masonry (CM) structures are vital in seismic design, utilizing tie-columns and tie-beams to augment the deformation capacity and structural integrity under lateral and gravity loads [38]. The behavior of RC frames incorporated with CM walls is significantly influenced by the interplay between the RC frames and the CM panels. This dynamic not only impacts the structural stability of the masonry walls, but also alters the failure mode of the RC frames, deviating from their original design expectations [39]. International codes provide foundational guidelines for confining elements, including specifications for longitudinal steel bars and tie sizes. However, these standards sometimes lead to seismic vulnerabilities, as evidenced by the weaknesses in CM structures revealed in past earthquakes. These include insufficient confining elements and masonry strength, a poor design, construction irregularities, and challenges associated with slender walls [40,41]. Studies that have analyzed earthquake damage in CM structures have commonly identified two primary failure modes: flexural and shear. These failure modes are influenced by a range of factors, such as the reinforcement percentage, the wall aspect ratio, the material strength, the confining element size, etc. [42,43,44,45,46,47]. When CM walls face in-plane lateral loads, they typically exhibit a diagonal tension shear failure pattern. This involves inclined cracks forming in the masonry walls, which then extend into the RC confining elements, eventually leading to failure [48].

Extensive research, both numerical and experimental, has been dedicated to developing shear and flexural strength criteria for CM structures, with a focus on the effects of confinement. Chourasia et al. [49] analyzed various masonry typologies, with confined masonry showing a superior lateral strength, ductility, and energy dissipation. Marinili et al. [50] investigated CM walls with varying tie-columns and found that tie-columns significantly enhance the ductility, initial stiffness, and seismic capacity. Kato et al. [51] analyzed the shear and axial reinforcement in CM tie-columns, noting an improved lateral load capacity with adequate axial reinforcement. They also suggested identifying an optimal axial-shear reinforcement ratio for further strength enhancement. Similar results were observed by Okail et al. [52] while exploring the structural response of life-sized CM walls with different axial and shear reinforcement ratios.

In a comparative study, Ashraf et al. [53] demonstrated that confined masonry, even when perforated by openings, offers almost double the seismic capacity of unreinforced masonry walls. Jorge et al. [54] assessed the in-plane flexural performance of CM piers, observing improved behavior with reduced reinforcement in the confining elements. Matosevic et al. [55] examined the connection details between masonry and tie-columns, noting enhancements in the hysteretic energy dissipation, seismic resistance, and wall stiffness due to confinement. Ahmed et al. [56] tested a full-scale confined masonry room under quasi-static loads and found that confinement nearly doubled the seismic strength compared to unreinforced structures. Understanding the actual structural behavior of existing masonry buildings is critical for conducting an accurate seismic assessment and developing conservation strategies. Full-scale testing plays a pivotal role in this context, as it provides invaluable insights that cannot be fully captured by numerical simulations alone. For instance, Gentile et al. [57] demonstrated the effectiveness of full-scale testing combined with finite element (FE) modeling in their study of an ancient tower. Their research utilized ambient vibration surveys to inform the FE model, leading to a more reliable seismic assessment of the structure. This approach highlights the necessity of integrating empirical data from full-scale tests to enhance the accuracy of structural evaluations and ensure the preservation of historical masonry buildings.

Quiroz et al. [58] conducted experiments on the cyclic behavior of CM panels with varying levels of reinforcement in confining components. Their findings indicated that, despite different reinforcement ratios, the seismic characteristics remained consistent except for walls with minimal reinforcement, which showed a reduced lateral strength. Ibrar et al. [59] varied the dimensions and steel amounts in confining elements, concluding that larger confining elements enhance the structural performance under cyclic loading, while the reinforcement ratio had a negligible impact on the cracking patterns and seismic capacity.

Recent advancements in CM construction have highlighted its potential for enhanced seismic resilience, particularly in earthquake-prone regions. However, a significant gap persists in understanding the behavior of CM under seismic conditions, primarily due to the variability in the design of and detailing practices for the critical confining elements. This variability, which is observed across different global guidelines and standards, challenges the prediction and optimization of CM structures’ seismic performance. Moreover, there is a notable deficiency in comprehensive research addressing the combined effects of reinforcement ratios and the dimensions of the confining elements on a CM wall’s seismic behavior. This study aimed to bridge these gaps by conducting an in-depth analysis of the in-plane seismic characteristics of CM structures, employing both experimental and advanced finite element methods. The novelty of this research lies in its systematic approach to quantifying the impact of varied confining element configurations on the seismic characteristics of CM walls. This approach is expected to contribute significantly to the development of more nuanced and effective design guidelines, thereby enhancing the safety and resilience of CM structures in seismically active areas.

## 2. Methodology

### 2.1. Experimental Setup and Instrumentation

#### 2.1.1. Description of Specimen

A confined masonry wall sample, previously studied by Ajmal et al. [38] under quasi-static reverse cyclic shear loading, was further examined in this research. The single-wythe pier specimen had dimensions of 1870 mm in length, 1850 mm in height, and 113 mm in width, maintaining an aspect ratio of approximately 1.0 to prioritize shear failure over flexural failure. A 50% scaled-down wall panel was constructed to fit within the operational limits of the testing facility, including the equipment capacities for load application and monitoring. The wall was built on an RC pad measuring 2470 × 300 × 300 mm (L × W × H), simulating a robust footing. To develop the steel reinforcement for the tie-columns, a steel cage was fixed in place before casting the footing beam, and the dowels were firmly embedded into the footing pad, following the guidelines of ACI 318-19 [60]. A 300 mm extension on each side of the footing beam was used to anchor the pier to the test floor, preventing sliding and uplifting. Additionally, instrumentation was installed to monitor any base movements.

**Figure 1:**
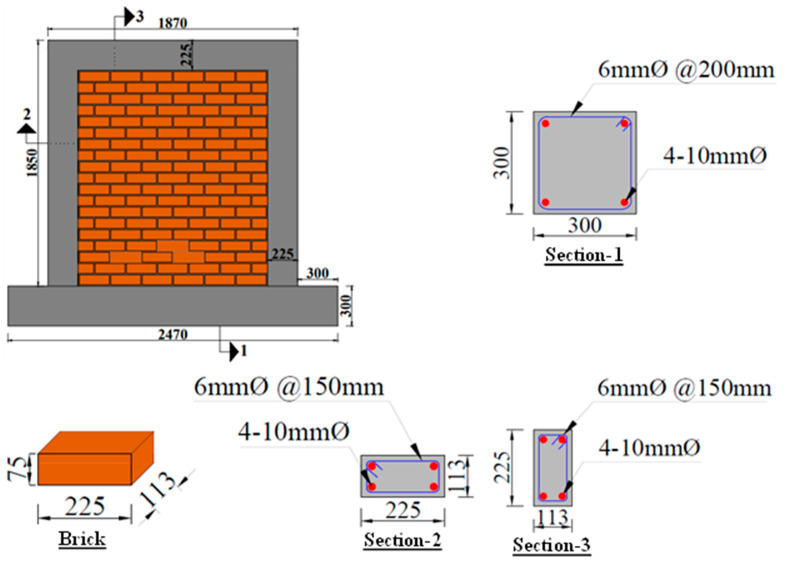
Diagrams and structural detailing of CM wall [38]. Red dots are steel reinforcement and blue lines are ties and stirrups. Numbers 1–3 are sections cut in column, beam and footing pad.

The masonry used standard clay bricks with a size of 225 × 113 × 75 mm (L × W × H), joined with 10 mm thick mortar joints. The bricks were pre-soaked to enhance mortar bonding. During the brickwork, half-brick toothing was incorporated on every alternate course of bricks to guarantee a tight fit of the masonry units with the confining elements. Tie-columns and tie-beam, each measuring 225 × 113 mm (width × depth), were constructed around the wall, with a depth matching the width of the wall. These confining elements were reinforced with four 10 mm deformed longitudinal steel rebars and 6 mm transverse reinforcement. The details of the steel reinforcement design and the geometry of the specimen are provided in Figure 1.

Before casting the confining elements, six strain gauges were affixed to the longitudinal and transversal steel bars of the columns and beams in the wall specimen to monitor the behavior of the reinforcement under a load. Strain gauges (10 mm long) were attached with a cyanoacrylate adhesive to the critical sections of the bars.

The thickness of the confining components was consistent with the masonry walls, standing at 113 mm. For the standard concrete, a mix ratio of 1:2:4 was adopted, consisting of 1 part ordinary Portland cement, 2 parts Lawrencepur sand, and 4 parts Sargodha crush. The slump for the beams was kept at approximately 85 mm, while for the columns, it was maintained at a higher value, roughly 110 mm, to facilitate the smooth pouring of concrete, especially into toothing, in the case of tie-columns. Special attention was paid to ensure the complete filling of the tooth gaps with concrete, and a vibrator was used to compact the concrete.

#### 2.1.2. Material Characteristics

The relevant standards were used to conduct experimental testing and determine the elastic modulus and strength characteristics of all the materials used in the confined masonry panels. This included mortar, brick, masonry, steel rebars, and concrete. The mortar mix design was 1:3 (cement–sand) with a water–cement ratio of 0.6. We used Lawrencepur sand, which has a fineness modulus (FM) value of 2.6, for both the mortar and the concrete, as determined through testing according to ASTM C136 [61].

To evaluate the compressive strength of the mortar, 50 mm cubes were tested, yielding a strength of 21.97 MPa with a coefficient of variation (COV) of 6.5%, in line with ASTM C109 [62]. The bricks’ compressive strength, determined as per ASTM C67 [63], was 19.74 MPa with a COV of 7.4%. For masonry, we tested a four-brick prism to find a compressive strength of 9.81 MPa and an estimated modulus of elasticity of 4700 MPa. This modulus was calculated from the secant line between points representing 5% and 33% of the compressive strength, as per the Masonry Standards Joint Committee 2011 [64].

The 28-day water-cured concrete cylinders showed a compressive strength of 31.37 MPa and an elastic modulus of 24,794 MPa, following ASTM C39/C39M [65] and ASTM C469/C469M-10 [66], respectively. The yield strength and elastic modulus of the 6 mm and 10 mm diameter steel rebars, estimated via direct tension tests, were 483 MPa (COV 2.1%) with a 188671 MPa modulus and 537 MPa (COV 1.3%) with a 191103 MPa modulus, respectively, which was compliant with ASTM A615 [67].

Finally, the diagonal compressive and shear strengths of the masonry, estimated by testing brick wallets and triplets measuring 600 mm × 600 mm and 225 mm × 245 mm, were 0.58 MPa (COV 13.8%) and 0.56 MPa (COV 11.2%), respectively, as per ASTM E519-02 [68] and BS EN 1052-3 [69].

#### 2.1.3. Instrumentation and Test Setup

**Figure 2:**
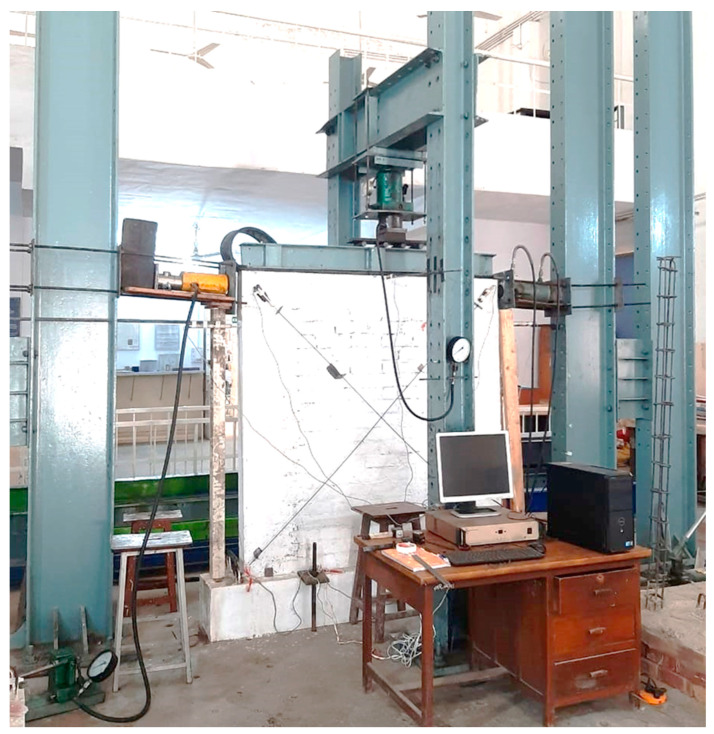
CM wall test setup and instrumentation [38].

The research experiment was carried out at the Test Floor Laboratory of UET Lahore, with the testing configuration depicted in Figure 2. The extended portion of the footing beam on both sides of the specimen was rigidly anchored to the strong test floor.

The beam–column joint was subjected to a pseudo-static reverse cyclic load along the in-plane direction. The lateral load was applied through hydraulic jacks placed on either side of the wall panel. Two 40-ton-capacity reaction frames were installed on both sides of the specimen with a steel I-section braced at the height of 1.5 m, on which hydraulic jacks were fixed to apply a lateral load to the specimen. Jack-I along with the load cell was meant to apply a push load to the specimen from left to right, whereas Jack-II along with the load cell was designated to apply a push load from right to left on the specimens. Load cells were utilized with hydraulic jacks to measure the lateral loads. The loading was applied at a small frequency of around 0.02 Hz.

**Figure 3:**
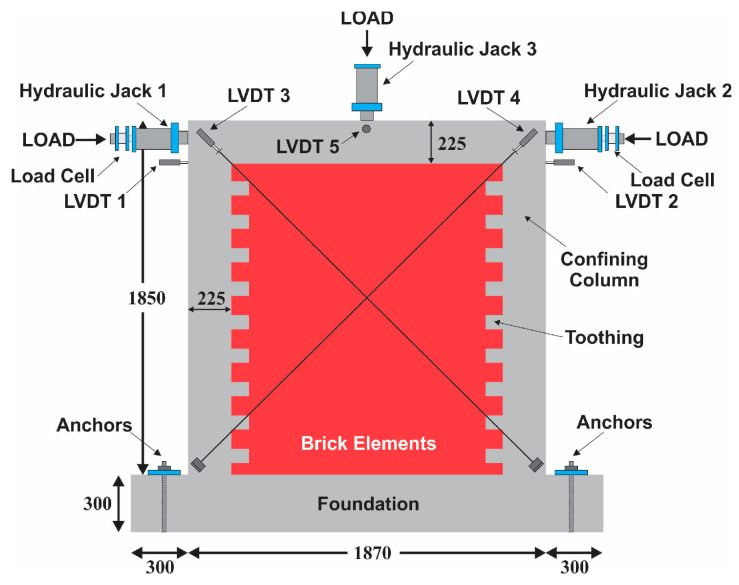
Schematics of test setup [38].

The measurement of in-plane, out-of-plane, and diagonal movement was conducted using a total of five LVDTs. To simulate the service roof load on the wall, an evenly spread gravity load of 0.35 MPa was constantly exerted by a hydraulic Jack-III on the specimen using a steel girder. To enable unimpeded lateral movement during loading, four rollers with a diameter of 50 mm were positioned on top of the steel girder. A strong packing was provided between the test specimen and the reaction frame to avoid specimen base slippage during loading. A schematic diagram of the experimental test setup is shown in Figure 3.

**Figure 4:**
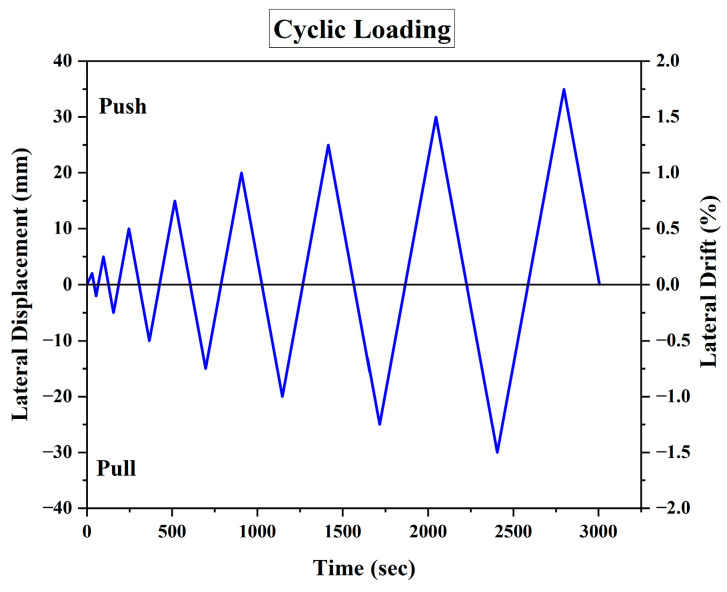
Time history of cyclic displacement with quasi-static loading [38].

Initially, a cyclic load was applied up to 2 mm of lateral displacement in the push-and-pull cycles; afterwards, the lateral loading continued until the failure of the specimens, conforming to ACI 374.1-05 [70], with a 5 mm increment in the lateral displacement after each cycle, as demonstrated in Figure 4. The test concluded when the specimen ceased to bear any additional loads and strain softening started, with a noticeable widening of the bed joint cracks in the case of the CM wall.

### 2.2. Numerical Modeling

A computer-based analysis was made by creating a 3D finite element model to simulate the CM wall tested experimentally. Also, the impact of the steel reinforcement ratio and the size of the confining elements on the seismic characteristics were studied numerically under pseudo-static reverse cyclic loading. For this purpose, the commercially available finite element software ANSYS 15.0 was used based on the past research on masonry walls [71,72,73,74,75,76,77,78].

#### 2.2.1. Modeling Technique

**Figure 5:**
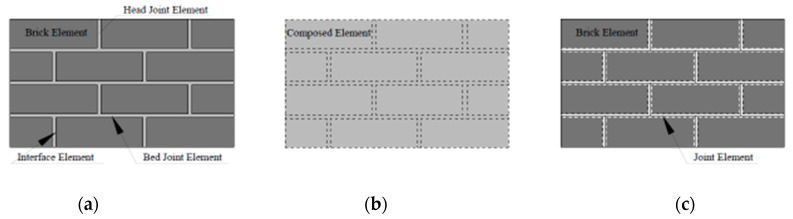
FEM techniques: (**a**) detailed micro-modeling, (**b**) macro-modeling, and (**c**) simplified micro-modeling.

Previous research has identified several numerical techniques for analyzing the structural performance of brick walls, which differ in their ability to predict failure mechanisms accurately. These techniques can be broadly classified into micro-modeling, macro-modeling, and simplified micro-modeling, as illustrated in Figure 5.

The detailed micro-modeling method is preferred when analyzing the maximum modes of failure (Figure 5a). Here, the bricks and mortar are modeled as continuum elements, while the brick–mortar contact surface is characterized by distinct elements showing discontinuities. Creating a separate model for each constituent element of the masonry structure (mortar, brick, and interface) with their unique properties demands additional data processing time in this kind of approach. On the other hand, the macro-modeling technique considers the entire brick wall as an identical element, and the wall panel is assigned distinctive properties in lieu of mortar and brick, while the mesh size remains identical to that of the brick (Figure 5b).

Although this approach requires less computational time, it is relatively approximate. Hence, this modeling technique is used when the degree of accuracy is not critical, such as in larger structures. Nonetheless, the simplified micro-modeling method preserves the basic wall configuration and dimensions, comprising the bricks and other essential features, such as detailed micro-modeling; however, it models the contact area of the interface elements and mortar joints as separate entities (Figure 5c). This modeling technique assigns the shear and compressive stress characteristics to the mortar using spring elements, while contact elements are used for the brick–mortar bond. It demands less computational time as compared to the detailed micro-modeling technique.

#### 2.2.2. Modeling

Various researchers have conducted FEAs to observe the complex behavior of brick walls and have shared their recommendations about the simplified methods [79,80,81,82,83,84,85]. The software ANSYS [86] utilizes 8-node, 3-degrees-of-freedom hexahedral elements to create replicas of the reinforced concrete and brick units in 3D, allowing for translational displacement in the x, y, and z directions at all nodes. The chosen element type, SOLID65, has the ability to crush under compression and crack under tension and can accommodate the inclusion of reinforcement rebar.

**Figure 6:**
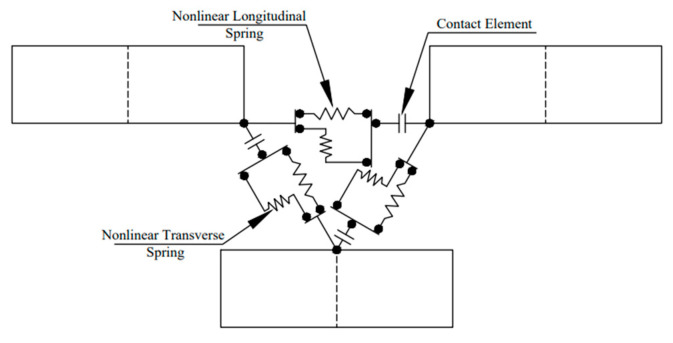
Brickwork joint specifications [38].

**Figure 7:**
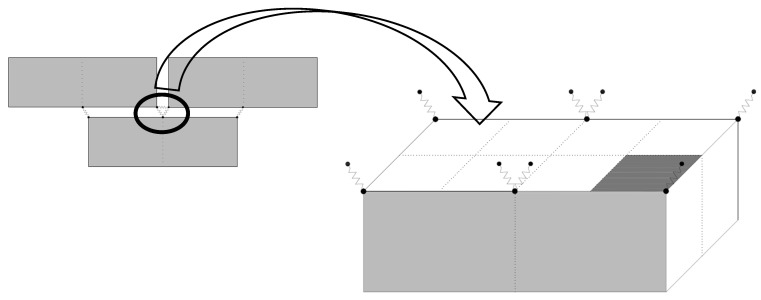
Tributary area on each node of spring element [38].

For numerical modeling, the distribution of steel rebar was assumed to be even and uniform through the concrete component. It is essential to define failure criteria to obtain the failure of a material. For steel rebar, the LINK180 element was used, which is a uniaxial tension-compression element with three degrees of freedom at each node: translational displacement in the nodal x, y, and z dimensions. To represent the behavior of the mortar joint, two nonlinear spring elements, COMBIN39, were employed in parallel, with a single contact element, CONTA178, connected in series to the spring elements, as shown in Figure 6. The axial and shear behavior of the mortar was simulated using the spring elements, while the bond between the mortar and brick was modeled using the contact element. The properties of both the contact elements and spring elements were established based on their respective material characteristics, with the inclusion of friction in the contact element. The force-deflection curve for these elements was defined using the stress-strain curve, with the force expressed as the product of stress and the tributary area over the node, as shown in Figure 7. The deflection of the joint was calculated as the product of the strain and the length of the spring. The length of the nonlinear spring element was defined as 90% of the joint thickness, while the dimension of the contact element was set at 10% of the joint thickness.

The brick elements were halved vertically to enable connections with courses above and below, as shown in Figure 7. To represent the connection between the bricks and mortar, including the mortar joint and bond, two parallel spring elements and one series contact element were used to connect the nodes of two bricks. The properties were assigned to two nonlinear spring elements to demonstrate the axial and shear behavior of the mortar, whereas the contact elements were allocated to represent the bond between the brick and mortar, with associated properties related to mortar and friction. The confining elements of the wall were simulated using the SOLID65 element, and the reinforcement was integrated into the concrete using the LINK180 element. To restrain the out-of-plane displacement of the test wall, the analysis included the z-directional restraint of all the wall nodes. Additionally, the bottom-most nodes of the test specimen were constricted in all directions to ensure fixed support at the bottom.

The overall properties used in the numerical modeling of confined masonry comprise those provided in Section 2.1.2. Some additional characteristics used in the simulation of CM walls are presented in Table 1.

## 3. Results and Discussion

### 3.1. Experimental Results

#### 3.1.1. In-Plane Load vs. Deformations

**Figure 8:**
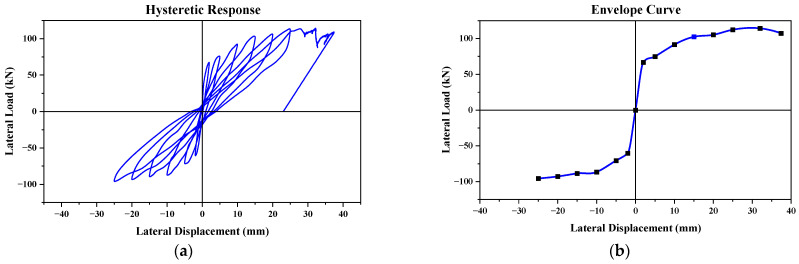
CM wall: (**a**) hysteretic behavior and (**b**) envelope curve.

The relationship between the in-plane load and displacement for the tested CM wall sample is depicted in Figure 8. The response of the wall panel demonstrated almost proportional cycles during both the compression and tension phases. With each subsequent hysteresis, the seismic resilience of the wall sample showed an upward trend, peaking at 114.3 kN, before receding to 109.1 kN in the final push load. These correspond to drift indexes of 1.73% and 2.02%, respectively. After reaching the highest strength capacity, the wall panel displayed a notable decline in seismic resilience, leading to the conclusion of the test. This pronounced drop was especially evident at higher drift levels, which was attributed to the confining forces. The lateral force spread across the cross-section of the masonry, A_w_, resulted in shear stress (V_max_/A_w_) of 0.67 MPa in the masonry segment. The initial rigidity (K_co_) of the wall stood at 35 kN/mm, and it had an overall energy dissipation measure of 5861 kN-mm.

The envelope curve (Figure 8b) derived from the peak points of the hysteresis loops for the wall under lateral cyclic loading served as a pivotal visual tool for interpreting the seismic performance of the wall. The rough symmetry of the curve around the y-axis denotes that the wall demonstrated analogous resistance behavior against push-and-pull lateral loads. At the outset, particularly between no displacement and minimal displacement, the wall showcases a pronounced initial stiffness. This is evident in the sharp escalation in load values with only minor increases in the displacement.

As we traversed further along the curve, a progressive flattening became discernible, particularly between the drift displacements of 15 mm and 32 mm in the push load. This change in the gradient of the curve highlights the inherent ductility of the wall, allowing it to sustain considerable deformations without experiencing a noticeable decline in strength. The peak values of the curve located around 112.3 kN and 98.4 kN, in the last push-and-pull load (i.e., the sixth and seventh cycles), encapsulate the peak resistance of the wall against lateral loads. The regions of the curve where a plateauing effect was noted, around the drift displacements of 32 mm and 25 mm, can potentially be ascribed to the yielding phenomenon of the masonry wall. Beyond these regions, the wall exhibited a tendency to experience heightened displacements with only marginal load alterations, suggesting potential structural vulnerabilities such as cracking or crushing. The slight decline in the load resistance observed at a displacement of 37.4 mm further corroborates this post-peak behavior. In essence, this envelope curve not only elucidated the masonry seismic response of the wall, but it also offered invaluable insights into its post-peak behavioral nuances. The cyclic responses of the experimentally tested specimen are presented in Table 2.

**Figure 9:**
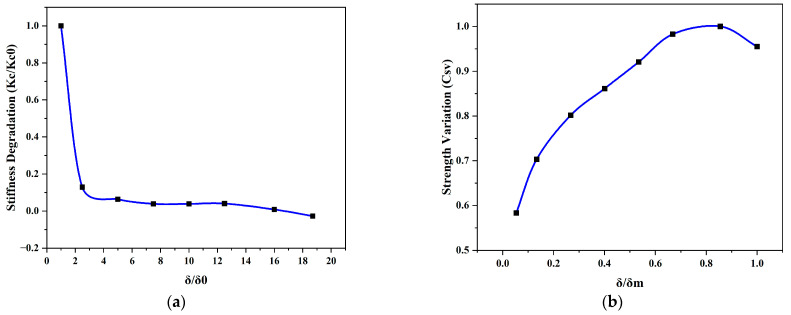
(**a**) Stiffness degradation; (**b**) strength variation. Blue line is graph by connecting the data points presented by black dots.

The degradation of the stiffness (K_c_/K_c0_) at each drift level was plotted against the drift δ, normalized with respect to the drift level in the first loading cycle, δ_0_ (Figure 9a). Additionally, the strength variation factor (C_sv_ = V/V_max_) was determined by normalizing the lateral loads against different drift levels with V_max_ and charted against δ/δ_m_ (Figure 9b). The resulting plot indicated an increase in the strength of the wall because of the presence of confining components in the panel.

#### 3.1.2. Steel Strain in Longitudinal Bar

**Figure 10:**
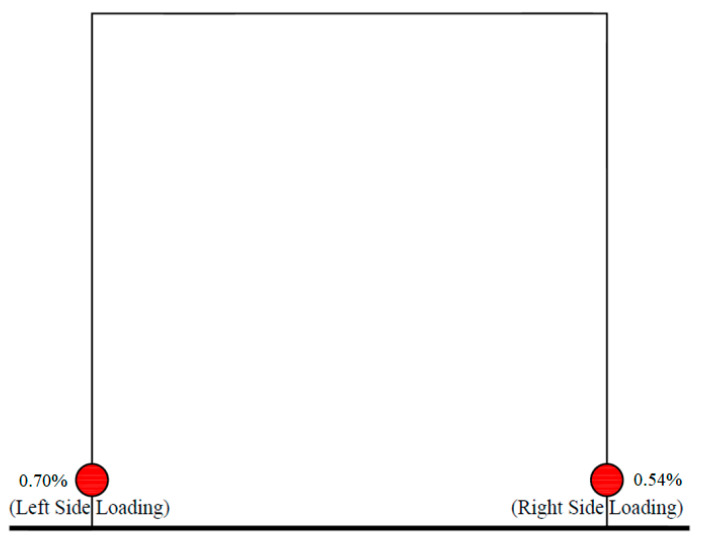
Drift index at steel yield in tension of OPC-CM.

Strain gauges, strategically placed at the base of each tie-column in the wall, recorded pronounced strains due to the yielding of the longitudinal steel. Figure 10 showcases the minimal lateral drift index values corresponding to the yielding of the reinforcement bars, aligning with the hysteretic behavior of CM. In the fourth push cycle, the longitudinal steel rebar of the left column indicated yielding at a 0.70% drift index, while during the 3rd pull cycle, the steel in the right column reached yielding at a 0.54% drift.

#### 3.1.3. Failure Mechanism

**Figure 11:**
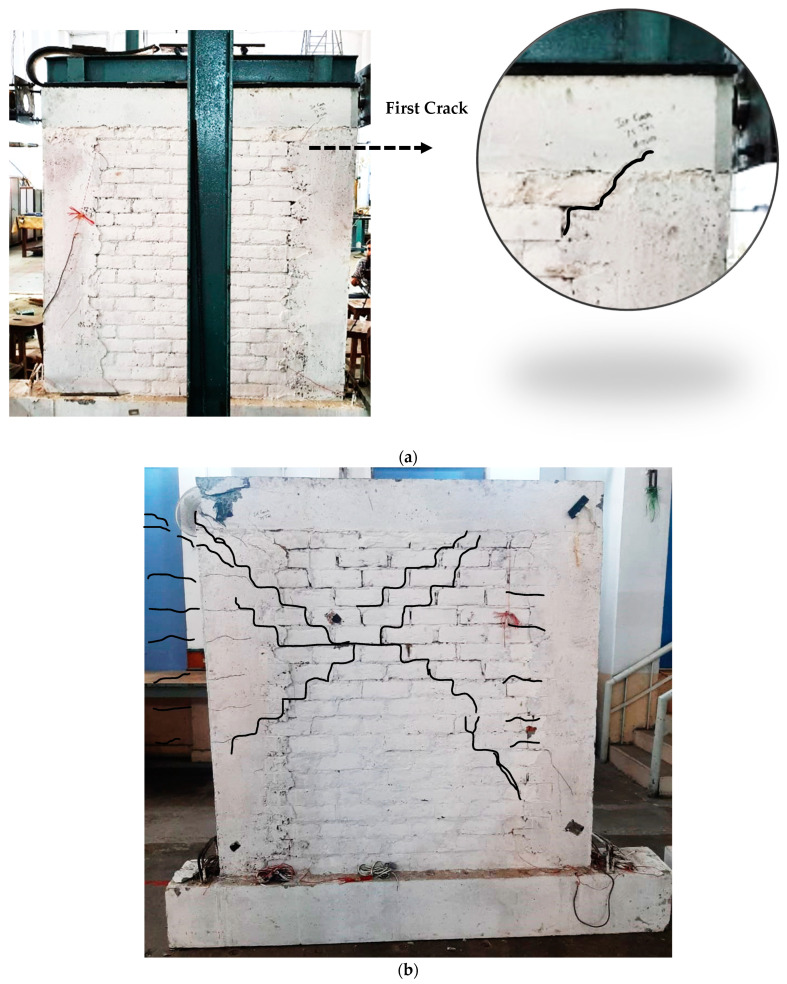
Crack pattern and damage observed in specimen: (**a**) first crack and (**b**) final state of damaged wall. Black lines are marker lines for cracks developed in specimen on being subjected by the lateral loads.

Figure 11 illustrates the cracking pattern and failure mode of the specimen wall. The initial crack appeared at the head joint of the masonry near the top at a 0.22% drift and a seismic load of 75.74 kN (Figure 11a). Increased loading led to downward crack propagation through the bed and head joints, with stair-stepped cracks emerging in the mortar at a 0.54% drift and 89.85 kN load. Diagonal crisscross cracks developed from the column–beam joint through the brick interface, reaching the opposite tie-column at a 1.08% drift and 103.21 kN load. The failure mode was characterized by shear failure in the confining columns and diagonal cracking in the masonry bed joint of the panel.

Confining components delayed the diagonal shear crack propagation from the wall to the tie-column, demonstrating a notable difference from infilled masonry structures. As the drift increased, flexural cracks in the tie-column appeared, leading to failure due to panel rocking once the tie-column reached its ultimate tension capacity at the base. The cracks predominantly followed the bed and head joints, with fewer cracks in the masonry units themselves.

In-plane failure in CM walls, which is critical due to its occurrence along the primary lateral load transfer path, often combines various damage modes. Shear-induced in-plane damage is common, presenting as bed joint sliding, diagonal compression, or tension, with shear cracks starting in the wall and extending to the tie-columns [87].

The wall panel reached its peak load-bearing capacity during the push-and-pull tests, with maximum values of 114.3 kN and 108.5 kN at 1.73% and 1.35% drift indexes, respectively. The failure occurred with an enhanced capacity and ductility, and was comparable to the findings by El-Diasity et al. [88]. The peak horizontal movement recorded was about 37.4 mm. The test concluded at the collapse prevention (CP) level as per ASCE/SEI 41-06 [89], with the diagonal shear cracks measuring up to 24 mm and flexural cracks in the tie-column of up to 9 mm wide. The damage was primarily localized to the upper third of the wall.

### 3.2. Numerical Simulation

In civil engineering, numerical modeling is essential for studying structural behavior in materials and designs. It is pertinent to validate these models with empirical tests to ensure accuracy. However, limitations exist, such as accurately representing complex materials such as masonry, concrete, and steel, and their interactions. Modeling crack growth and damage under repeated loads is difficult and may not fully reflect real-world behaviors such as energy loss and stiffness reductions. Computational limits can restrict the model detail, and oversimplifications may impact the accuracy. Replicating real-world load and boundary conditions is also challenging. Validating models with experimental data is vital, but can face data constraints, leading to differences between the simulated and actual outcomes.

#### 3.2.1. Validation of Numerical Wall Model

**Figure 12:**
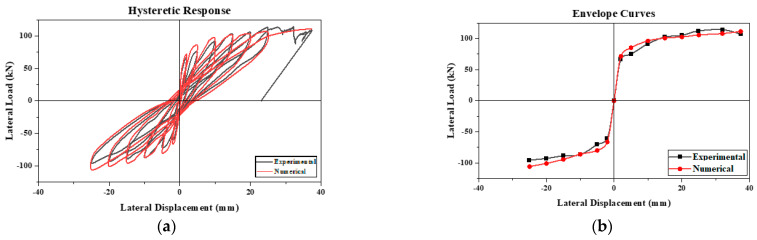
Validation of CM wall: (**a**) hysteretic behavior and (**b**) envelope curve.

This paper presents a comparison between the experimental results and a numerical model of a confined masonry wall panel, which showed a good correlation in seismic capacity (V_num_) with a marginal difference of 2.6%. The hysteretic response of the numerical model initially mirrored the experimental results; however, as the lateral drift increased, the model showed enhanced energy dissipation, as depicted in Figure 12. The envelope curves from the simulation confirmed the experimental observation that a higher lateral drift leads to increased lateral strength. Additionally, the initial stiffness derived from the model closely matched the experimental results, with a slight deviation of only 5.4%. In representing structural damage, the stress contours were plotted in ANSYS, as the graphical user interface of the software has limitations in visualizing bed joint cracks in the masonry pier. Despite this constraint, the finite element model exhibited a commendable degree of accuracy in reflecting the damage observed during the experiment.

**Figure 13:**
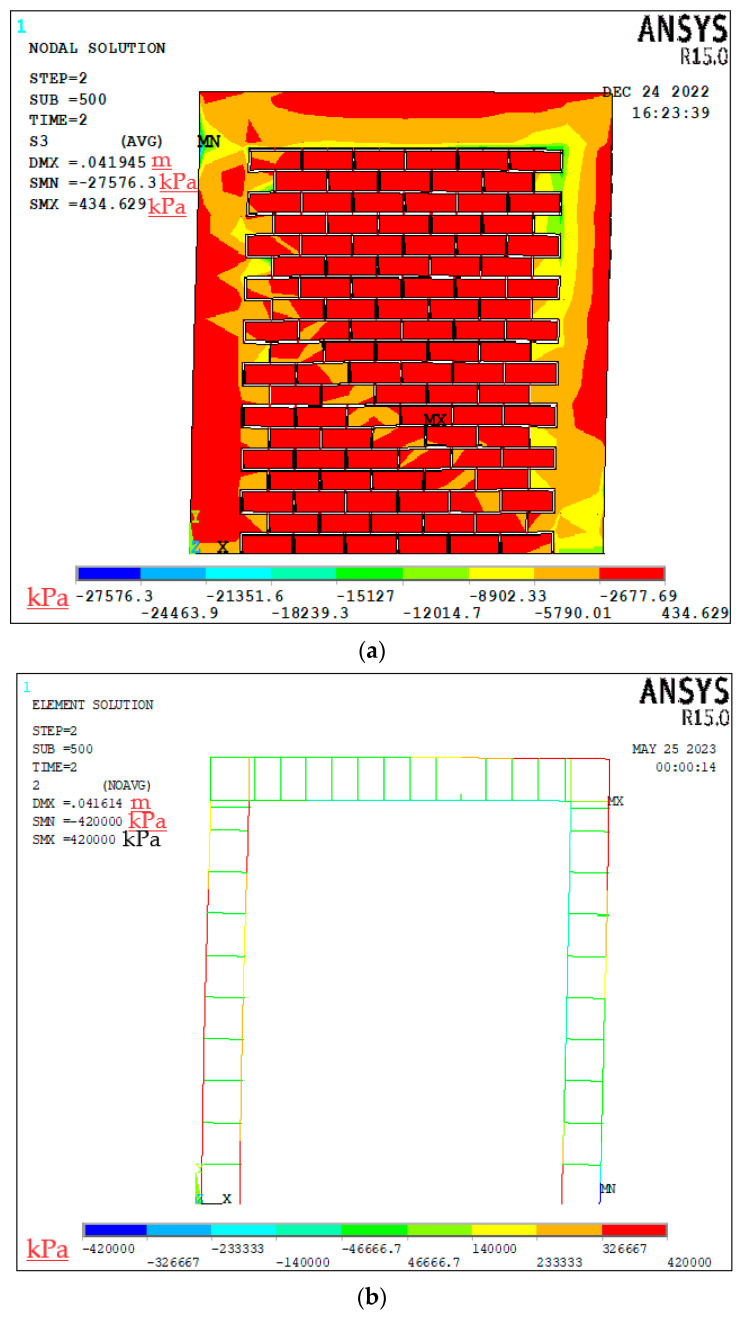
CM wall: (**a**) stress distribution in wall panel and (**b**) stress distribution in steel rebars.

Figure 13a displays the stress distribution of the wall panel at its breaking point, revealing distinct diagonal stress patterns indicative of shear failure. This shear failure pattern was characterized by the formation of diagonal cracks, which are typical in masonry walls subjected to lateral loads. This observation was consistent with the failure mode seen in the actual wall test, validating the accuracy of the finite element (FE) model in capturing the primary failure mechanism.

The FE model of the CM wall showed peak compressive stress at the beam–column intersection on the confining column, a critical location where the wall ultimately failed. This concentration of stress suggests that the junction of the beams and columns in the confining frame is a vulnerable point and is prone to experiencing significant stress during seismic events.

During the loading and unloading cycles, the principal stresses varied significantly, illustrating the dynamic response of the wall to cyclic loads. Although the FE model could not accurately represent bed joint fractures—an inherent limitation of the model—it closely approximated the experimental damage. This approximation was evidenced by the stress distribution patterns generated in ANSYS, which aligned well with the observed damage in the physical tests.

Under cyclic loading, the steel rebars in the tie-columns yielded before the failure of the wall, as depicted in Figure 13b. The yielding of the rebars was an important aspect of the wall’s performance, as it indicates that the tie-columns are effectively dissipating energy and undergoing plastic deformation prior to the overall failure of the structure. The stress in the longitudinal steel rebars of the tie-columns reached and was limited to the yield point, due to the elasto-plastic constitutive model of steel used in the modeling. This behavior highlights the role of the steel rebars in providing ductility to the wall system, allowing it to undergo significant deformation without sudden failure. The FE model’s ability to simulate the yielding of steel rebars and the subsequent stress distribution provided valuable insights into the performance of CM walls under seismic loading. The stress patterns and failure modes aligned closely with experimental observations, demonstrating the model’s reliability in predicting the structural response of CM walls.

#### 3.2.2. Parametric Study

A masonry wall panel, featuring 225 mm deep confining elements and No. 10 longitudinal reinforcement, was subjected to reverse cyclic loading to assess its seismic behavior. This was then compared with a numerical model. The outcomes from both the experimental and numerical approaches showed a good match within acceptable limits, as illustrated in Figure 12. This study was expanded to explore the effects of varying the reinforcement ratios and confining element sizes on the seismic performance of the wall. To achieve this, the depth of the confining elements was varied, creating combinations of each depth with three distinct reinforcement ratios to examine their collective impact. The dimensions of the confining elements and the diameter of the longitudinal bars for nine different confined masonry walls, including the control specimen, are detailed in Table 3. The term “CM” refers to confined masonry, with the initial number indicating the size of the confining elements in millimeters and the subsequent number denoting the size of the longitudinal reinforcement within these elements.

**Figure 14:**
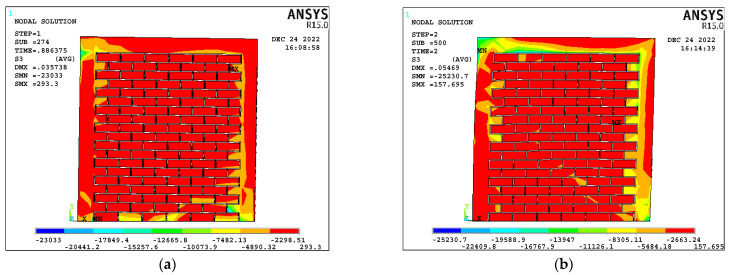

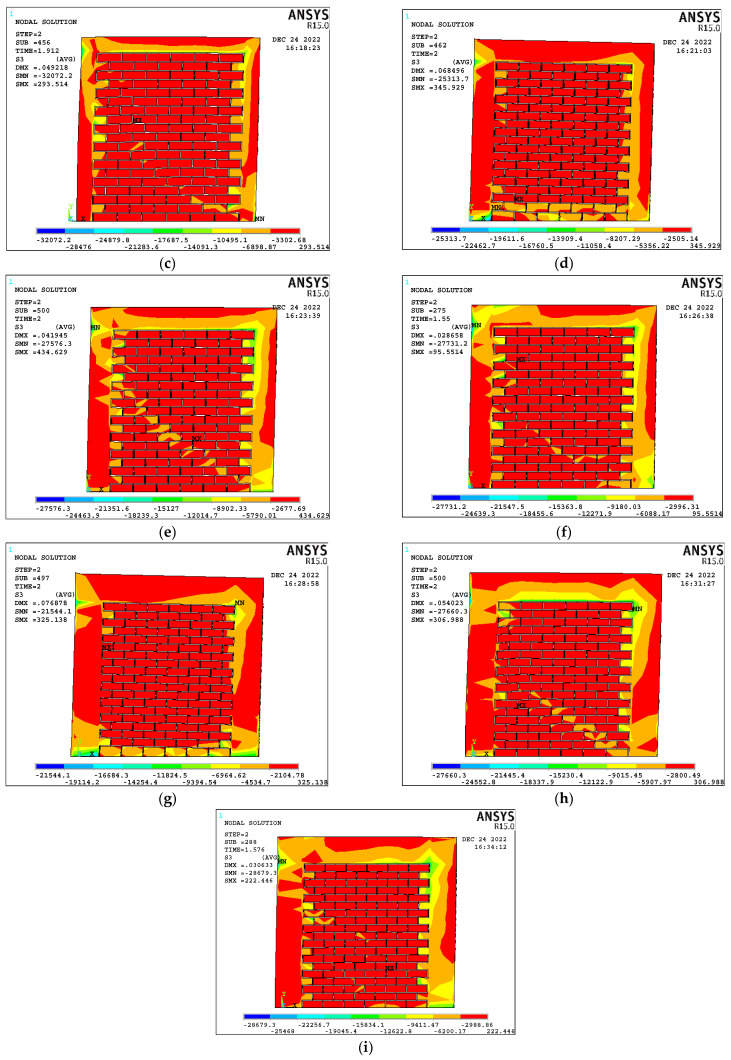
Damage shape contour diagrams of (**a**) CM-150-6, (**b**) CM-150-10, (**c**) CM-150-13, (**d**) CM-225-6, (**e**) CM-225-10, (**f**) CM-225-13, (**g**) CM-300-6, (**h**) CM-300-10, and (**i**) CM-300-13. MX means maximum stresses and MN means Minimum stresses.

Figure 14 displays the stress contour plots of all the wall panels at the point of failure, where it is evident that shear failure was common across all panels. In most cases, the walls exhibited peak principal compressive stresses in the confining columns, either at the base or near the load application point, occurring after the concrete had reached its maximum compressive strength. The principal stresses in the models fluctuated during the push–pull loading and unloading cycles. Due to limitations of the FE model, crisscross cracks could not be modeled; however, smeared cracks were produced in the models.

**Figure 15:**
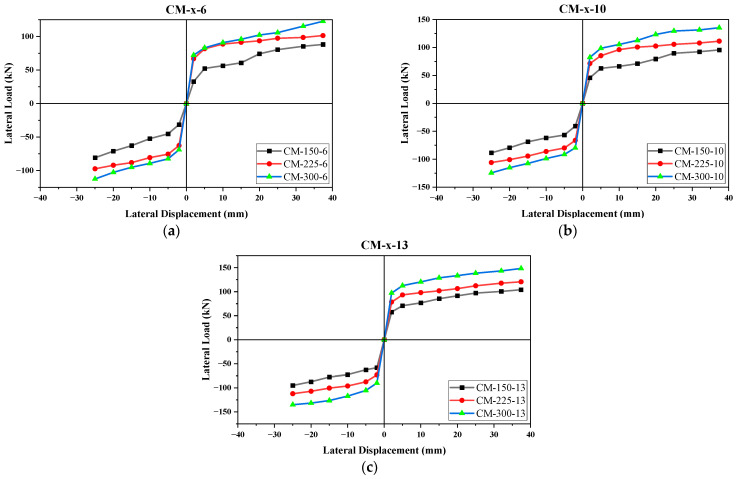
Envelope curves for same reinforcement size: (**a**) CM-x-6, (**b**) CM-x-10, and (**c**) CM-x-13.

**Figure 16:**
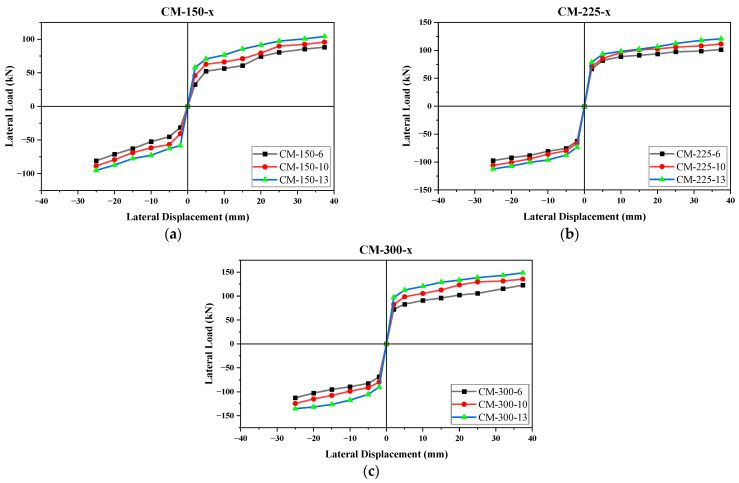
Envelope curves for same confining element size: (**a**) CM-150-x, (**b**) CM-225-x, and (**c**) CM-300-x.

The envelope curves given in Figure 15 and Figure 16 evidently illustrates that both the steel reinforcing percentage and the size of the confining components have a considerable effect on the lateral load response. The maximum lateral load strength and the initial stiffness of the walls increased with an increase in the depth of the confining components and the diameter of longitudinal steel rebars. The nomenclature adopted for the envelope curves illustrated in Figure 15a–c symbolizes “CM” as confined masonry, “x” as the variable for the depth of the confining elements, and the number as the the size of longitudinal reinforcement in the confining elements.

The effect of the confinement size was evaluated in all the masonry walls by keeping the reinforcement the same. The percentage differences between the maximum lateral load carrying capacities of CM-225-6 and CM-300-6 with respect to CM-150-6 were observed to be 13.9% (push), 18.5% (pull) and 32.7% (push), 32.8% (pull), respectively (Figure 15a). As the dimensions of the confining elements increased, an increase was observed in the lateral capacity. Similarly, the lateral strength of CM-225-10 and CM-300-10 was found to be 15.1% (push), 17.9% (pull) and 34.3% (push), 33.7% (pull), respectively, and was more than that of CM-150-10 (Figure 15b). The same strength was 14.7% (push), 16.5% (pull) and 35.2% (push), 34.7% (pull) greater in CM-225-13 and CM-300-13, respectively, as compared to that of CM-150-13 (Figure 15c). Although the highest percentage increase in the lateral load strength was found in the case of CM-300-10 with respect to CM-150-10, the maximum lateral strength obtained was in the case of CM-300-13. From the envelope curves, it was found that the stress pattern was the same in all cases (Figure 15a–c), yet the maximum lateral load strength was observed to increase with an increase in the confinement size.

According to Hook’s law, the lateral stiffness is directly proportional to the moment of inertia of confining columns [90]. By increasing the size, the stiffness of the confining columns increased and, resultantly, the wall exhibited a higher lateral seismic load. The increase in the column stiffness caused the reduced transfer of strain in the masonry panel when subjected to a lateral load. Most of the energy was dissipated by the tie-columns and the rest was transmitted to the masonry panel, once the capacity of the column exhausts.

Jin et al. [91] studied the effect of the cross-section size on the flexural failure behavior of RC cantilever beams subjected to cyclic loading. Based on tests conducted on 15 RC beams, it was found that the maximum lateral load was increased to 84.5%, 134.7%, 160.2%, and 169.2% when the depth of the beam was increased from 200 mm to 400 mm, 600 mm, 800 mm, or 1000 mm, respectively. Jin et al. [92] conducted similar research and studied the effect of the cross-section size on the lateral load strength of RC columns subjected to cyclic loading. Based on experimentation on 12 square RC columns, the authors inferred that, by increasing the size of the columns from 300 mm to 500 mm and 700 mm, the peak lateral load increased by an average of 77% and 128%, respectively. Tajzadah et al. [93] carried out an analytical study on a G + 14 story RC buildings to find out the effect of column size on the overall stiffness and seismic response of a building subjected to ground shake. It was concluded that, by increasing the size of the reinforced concrete column from 500 mm to 700 mm, the base shear was increased by 6.4%. The lateral stiffness and ultimate strength of a wall panel are dependent on the relative stiffness of the column and infill material, but practically independent of the beam stiffness [94]. The certain reason behind this phenomenon is the establishment of a sound bearing of columns on the infill material through the toothing effect. Therefore, a combination of tie-columns with an increased size and an optimal size of bond beam may be more suitable for making the structure both stiffer and more cost-effective.

The nomenclature adopted for the envelope curves given in Figure 16a–c denotes “CM” as confined masonry, the first number as the depth of the confining elements, and “x” as the variable for the size of the reinforcement. The percentage difference of the maximum lateral load strengths of CM-150-10 and CM-150-13 with CM-150-6 were 7.6% (push), 8.9% (pull) and 16.5% (push), 16.2% (pull), respectively (Figure 16a). In view of the stress contour plots of damaged wall panels, the maximum stresses at the peak lateral load were low in CM-150-6 due to a small size at the time of reinforcement, whereas in CM-150-10 and CM-150-13, the stresses were observed to increase with an increase in the reinforcement size. The failure in all panels occurred due to the crushing of concrete.

As shown in Figure 16b, the difference between CM-225-10 and CM-225-13 with CM-225-6 is 9.4% (push), 8.4% (pull) and 17.3% (push), 14.3% (pull) respectively. Like the previous wall panels, the maximum stresses were observed in CM-250-13 due to the larger reinforcement size, and by decreasing it, the peak stresses were found to decrease, even with the same size of confinement.

The maximum lateral strengths in CM-300-10 and CM-300-13 were found to increase by 9.8% (push), 10.0% (pull) and 19.0% (push), 18.2% (pull), respectively, with respect to CM-300-6 (Figure 16c). The maximum increase in the peak lateral strength was noted in CM-300-13 due to a large size of reinforcement and confining elements, which evidently reflected the reinforcing role of the confining elements in a wall subjected to the seismic load.

Steel reinforcement was a major contributor to the ductility and deflection of the CM wall. An increase in the diameter of the steel bar increased the initial stiffness and lateral strength; however, the most important parameter was the reinforcement ratio, which had a direct influence on the ductility and resistance against tensile forces. Based on the load moment interaction curves, the moment capacity of the reinforced concrete column significantly increased with an increase in the reinforcement ratio [95], and the same was observed in this study. Wang et al. [96] made an experimental investigation on the effect of the reinforcement ratio on the capacity of RC columns to resist lateral impact loading. The difference in the reinforcement ratio resulted in a significant difference in the ultimate load capacity. The maximum improvement observed was a 24.7% increase in the lateral capacity by increasing the reinforcement (two No. 18 bars) to three No. 18 bars in a 250 × 300 mm RC column. Pham et al. [97] experimentally and numerically investigated the sensitivity of the lateral impact response of RC columns reinforced with GFRP bars and stirrups to the reinforcement ratio. Based on the experimental results, the impact force was observed to linearly increase with an increase in the reinforcement ratio in RC columns. The lateral impact load capacity improved by 33.8%, 53.6%, and 46.4% when increasing the reinforcement ratio from 0.64% to 1.23%, 2.02%, and 2.89%, respectively, under an impact at 30° inclination. The slight decline in the impact force value at a 2.89% reinforcement ratio was attributed to an error in measuring the impact force.

Chrysanidis [98] examined the stability of scaled-down reinforced concrete seismic walls (1:3 scale) under varying tensile and compressive loads. Using specimens with low (1.79%) and high (3.19%) longitudinal reinforcement ratios, the results showed that increased reinforcement ratios improve the seismic resistance of walls for all levels of elongation. Chrysanidis [99] also investigated the effect of maximum-code-prescribed reinforcement ratios (4.02%) in reinforced concrete seismic walls, revealing that, even at this high reinforcement level, walls are susceptible to lateral buckling under certain tensile strains. This highlights a crucial gap in understanding the role of reinforcement ratios in seismic wall stability. In a further study, Chrysanidis [100] focused on the effect of the reinforcement ratio on the seismic response of RC walls modeled using prism elements. In the study, 22 tests were conducted using 11 column specimens with varying reinforcement ratios (from 1.79% to 10.72%), constructed at a scale of 1:3 to simulate the boundary edges of the structural walls. The experimental results illustrated an improvement in the ultimate failure load capacity of the columns by 112% with an increase in the reinforcement ratio.

It is pertinent to mention that, although the reinforcement ratio in wall panel CM-300-x was less than those of panels CM-150-x and CM-200-x, when compared with the same size of reinforcement, the initial stiffness and maximum seismic load were higher in the case of CM-300. This means that the reinforcement ratio can affect the ductility and deflection of a wall, but the seismic properties, including the initial stiffness and the ultimate load capacity, are governed majorly by the size of confining elements.

The structural behavior is similar among all types of panels, but it has been clearly illustrated that the size of reinforcement and the confining elements are proportional to the seismic properties (initial stiffness, maximum lateral load strength, energy dissipation, etc.).

## 4. Conclusions

This research entailed an experimental and numerical program focused on assessing the seismic performance of a single-wythe CM wall with an aspect ratio of approximately 1.0, subject to pseudo-static reverse cyclic loading controlled by displacement. Key aspects such as the lateral load capacity, the maximum drift, the initial stiffness, failure mode, stiffness degradation, and the damage pattern were thoroughly examined. Complementing this, an FE analysis was conducted for result validation and comparison. This study also explored the lateral stiffness of confined masonry walls, particularly investigating the influence of the confining element size and the steel percentage. This was achieved through a numerical parametric study, where the depth of the confining elements was varied alongside three different reinforcement sizes, allowing for an analysis of their combined effects. The significant findings from this research include the following:

1. The hysteretic response of the masonry panel exhibited approximately symmetric loops in push-and-pull load cycles with an increasing trend of lateral strength except in the last push load, in which the load carrying capacity reached maximum value of 114.3 kN and then decreased to 109.1 kN, corresponding to 1.73% and 2.02% drift index, respectively.
2. The shear failure mode observed in the test specimen demonstrated that the toothed contact surface between the masonry panel and the tie-column remained intact, providing evidence of the superior performance of the CM wall compared to the infilled structures.
3. The width of the diagonal shear cracks was noted as 24 mm, while the width of the largest flexural crack in the tie-column was measured to be 9 mm.
4. The FE model of the experimentally tested wall panel was developed based on a simplified micro-modeling technique, with the same parameter values offered an approximate concordance with the experimental results by a maximum error of 2.6% for ultimate lateral load strength and 5.4% for initial stiffness.
5. The parametric study indicated a positive correlation between the seismic strength of confined masonry walls and the size of both the confining components and the longitudinal reinforcement. An increase in the size of the confining elements enhances the moment of inertia, leading to a higher lateral load capacity. Additionally, a greater column stiffness results in reduced deformation transfer to the masonry panel under lateral loads. The tie-columns primarily dissipate energy, with the remaining load transmitted to the masonry panel once the columns reach their capacity.
6. Enhancing the size of the steel reinforcement bars improved the seismic strength of the structure. However, the reinforcement ratio emerged as a critical factor, directly influencing the ductility and tensile resistance. The load–moment interaction curves demonstrated that the moment capacity of the reinforced concrete columns increases substantially with higher reinforcement ratios, a finding corroborated by this study.

It can conveniently be summarized from the numerical and experimental studies that the confined masonry wall exhibited considerably improved seismic characteristics without the separation of the toothed interface between the confining columns and the masonry panel, endorsing a significant difference between the infilled and confined masonry structures. Also, the increment in the size of the confining elements and the reinforcement ratio resulted in a greater lateral load capacity and stiffness of the wall. The proposed study can be adopted for perforated confined masonry walls to compensate for the loss of wall stiffness due to the openings. The future scope of this study includes the extension of the present parametric study to find a combination of the optimal size of the confining elements and the reinforcement ratio to make the structure both stiffer and cost-effective. The numerical and experimental findings presented in this study can serve as a basis for further testing on confined masonry walls with varying aspect ratios, masonry strengths, and panel numbers.

## Figures and Tables

**Table 1:** Additional data values for numerical modeling.

Properties	Assigned Values
Poisson’s ratio for bricks: νb	0.20
Poisson’s ratio for mortar: νm	0.15
Poisson’s ratio for concrete: νc	0.16
Normal stiffness of CONTA178 element: Kn	13,967,223 kN/m
Tangential stiffness of CONTA178 element: Ks	6,072,705 kN/m

**Table 2:** Cyclic responses of experimentally tested specimen [38].

V_cr_ (kN)	V_max_ (kN)			V_max_/A_w_ (MPa)	δ_m_ (%)			E_d_ (kN-mm)
	**Push**	**Pull**	**Avg**		**Push**	**Pull**	**Avg**	
75.7	114.3	98.2	106.2	0.67	2.0	1.4	1.7	5861

**Table 3:** Specifications of CM walls modeled for parametric study.

Specimen Name	Nomenclature of CM Walls	In-Plane Dimension of Confining Elements (mm)	Size of Longitudinal Rebar (mm)	Steel Reinforcement Ratio (%)
1	CM-150-6	150	6	0.17
2	CM-150-10	150	10	0.46
3	CM-150-13	150	13	0.78
4	CM-225-6	225	6	0.11
5	CM-225-10	225	10	0.31
6	CM-225-13	225	13	0.52
7	CM-300-6	300	6	0.08
8	CM-300-10	300	10	0.23
9	CM-300-13	300	13	0.39

## Data Availability

The original contributions presented in the study are included in the article, further inquiries can be directed to the corresponding author.
